# Neighbourhood cohesion, loneliness and perceived social isolation prior and during the COVID-19 pandemic. Longitudinal evidence from the German Ageing Survey

**DOI:** 10.1007/s00127-023-02447-7

**Published:** 2023-03-13

**Authors:** André Hajek, Benedikt Kretzler, Carolin Walther, Ghazal Aarabi, Larissa Zwar, Hans-Helmut König

**Affiliations:** 1 Department of Health Economics and Health Services Research, University Medical Center Hamburg-Eppendorf, Hamburg Center for Health Economics, Martinistr. 52, 20246 Hamburg, Germany; 2 Department of Periodontics, Preventive and Restorative Dentistry, Center for Dental and Oral Medicine, University Medical Center Hamburg-Eppendorf, Hamburg, Germany

**Keywords:** Neighbourhood cohesion, Loneliness, Social isolation, Social exclusion, Social contact, Social embeddedness

## Abstract

**Purpose:**

To investigate the longitudinal association between neighbourhood cohesion and loneliness as well as perceived social isolation prior and during the COVID-19 pandemic (stratified by sex).

**Methods:**

Longitudinal data were taken from a nationally representative sample (German Ageing Survey) of inhabitants aged 40 years and over in Germany prior (wave 6: year 2017) and during the COVID-19 pandemic (wave 8: November 2020 until February 2021; *n* = 6688 observations, mean age was 67.4 years). The De Jong Gierveld tool was used to measure loneliness and the Bude and Lantermann tool was used to measure perceived social isolation. Neighbourhood cohesion was assessed based on different items.

**Results:**

FE regressions showed that decreases in closeness of contact with neighbours were associated with increases in loneliness and perceived social isolation levels among men, but not women. In contrast, decreases in different indicators of involvement in neighbourhood activities were associated with increases in loneliness and perceived social isolation levels among women, but not men.

**Conclusion:**

Changes in neighbourhood factors are differently associated with loneliness and perceived social isolation among middle-aged and older women and men. Gender-specific efforts to avoid loneliness and social isolation are, therefore, needed.

## Introduction

Prior research has clearly demonstrated that loneliness and perceived social isolation are frequent among individuals in the second half of life in Germany prior [1] and particularly during the COVID-19 pandemic (e.g., due to social distancing) [2]. Both, loneliness and perceived social isolation are of great importance, because they are associated with chronic conditions and premature mortality—and can avoid successful ageing [3, 4, 5]. Thus, understanding the determinants of loneliness and perceived social isolation is relevant. 

Recent systematic reviews clarified the factors associated with loneliness and social isolation [6, 7]. For example, these reviews showed that being married is a protective factor against loneliness and social isolation [6, 7]. While several of these sociodemographic determinants of loneliness and social isolation have been clarified among middle-aged and older adults, only a few studies exist investigating the association between neighbourhood-related factors and loneliness as well as perceived social isolation [8, 9, 10, 11, 12, 13]. For example, based on data from Hongkong, a previous cross-sectional study showed an association between higher neighbourhood social cohesion and lower loneliness levels among older adults prior to the COVID-19 pandemic [8]. In contrast, based on cross-sectional data from the Netherlands prior to the pandemic, another former study did not find an association between objectively measured social and physical neighbourhood characteristics and loneliness among older adults [9]. Former research also demonstrated an association between living in a deprived area and higher social exclusion in later life prior to the pandemic [11]. Another cross-sectional study showed higher isolation levels among urban residents with disabilities (compared to rural residents with disabilities) [13]. Similarly, an association between perceived neighbourhood built environment (“human-made space in which people live, work and recreate on a day-to-day basis” [14]; for example: green spaces, parks, traffic flow, etc.) and lower loneliness levels have been demonstrated among older European adults prior to the pandemic [12]. However, these aforementioned studies are clearly restricted in their cross-sectional design which makes it difficult to clarify the directionality. One longitudinal study based on data from the Health and Retirement Study showed that increases in perceived trustworthiness and helpfulness of neighbours were associated with decreases in loneliness from the year 2010 to the year 2014 [10].

Due to the limited knowledge based on longitudinal data, the aim of this longitudinal study was to examine the association between neighbourhood cohesion and loneliness as well as perceived social isolation prior and during the COVID-19 pandemic. Such knowledge about a potential association between neighbourhood cohesion and loneliness as well as perceived isolation among individuals in their second half of life is of great importance to identify individuals at risk for high loneliness and isolation levels longitudinally. One step further, it may assist in avoiding high loneliness and isolation levels by strengthening neighbourhood cohesion.

We assume that the cohesion in the neighbourhood may have suffered, in particular due to the social distancing [15] during the partial lockdowns in Germany. A poor neighbourhood cohesion may contribute to feelings of not belonging to the society or may contribute to a gap between actual and desired social relationships [8]. Therefore, we hypothesize:

- A poor neighbourhood cohesion is associated with higher loneliness levels
- A poor neighbourhood cohesion is associated with higher perceived social isolation levels

## Methods

### Sample

Data were taken from wave 6 and 8 of a sample including community-dwelling inhabitants aged 40 years and over (i.e., second half of life; “German Ageing Survey”, short: DEAS). This study started in 1996 and is funded by the Federal Ministry for Family Affairs, Senior Citizens, Women, and Youth (BMFSFJ). It has a cohort-sequential fashion meaning that new baseline samples were mostly introduced every 6 years (i.e., in wave 2, year 2002; in wave 3, year 2008; in wave 5, year 2014), whereas pure panel assessments were conducted in wave 4 (year 2011), wave 6 (year 2017), and wave 8 (November 2020 to February 2021).

Individuals were recruited using a national probability sampling. Various topics were covered in the DEAS study such as family situation, economic issues, health-related factors, or transition to old age. In a first step, individuals were interviewed (in wave 8 only by phone in the CAPI field due to the COVID-19 restrictions; a pretest was conducted in August 2020), for example referring to sociodemographic issues. In a second step, the participants were given a questionnaire to fill out additionally (including quite sensitive factors such as psychosocial factors). For example, the response rate was 65% in wave 8. The mean interview duration equaled about 75 min in this wave. Refusal and health restrictions were the most frequent causes for lack of follow-up data. Klaus et al. gave further details regarding the DEAS study in general [16].

For reasons of data restrictions, we focused on wave 6 and wave 8. Since we used linear fixed effects (FE) regressions in our current study, individuals were only included in our analytical sample when they participated both, in wave 6 and wave 8 and had changes in the variables examined over time. Therefore, our analytical sample equaled 6688 observations when perceived social isolation was used as outcome (which corresponds to 3344 individuals, because we used two waves (6688 divided by 2)). Stratified by sex, 3344 observations (which corresponds to 1672 individuals) were present in the analytical sample among men. Equally, 3344 observations (which corresponds to 1672 individuals) were present in the analytical sample among women.

The Declaration of Helsinki was followed when conducting the DEAS study. It should be emphasized that the DEAS study did not require an ethical declaration because the prerequisites for such a statement were not satisfied (risk for the respondents, lack of information about the aims of the study, examination of patients). Based on the recommendation of a standing council of the DEAS that determined no ethics vote was required, the German Centre of Gerontology, which is responsible for the DEAS study, did not apply for an ethics vote.

### Dependent variables

As outcomes, we used loneliness and perceived social isolation in our study. Loneliness was assessed using the De Jong Gierveld loneliness instrument—which has six items (four levels each) [17]. One example is: “I miss the pleasure of the company of others”. A mean score was calculated (using all four items: from 1 to 4) whereby higher scores indicate higher loneliness levels. In wave 6, Cronbach’s alpha was 0.83 (McDonald’s omega: 0.84) in our study. In wave 8, Cronbach’s alpha was 0.80 (McDonald’s omega: 0.81) in our study.

An instrument developed by Bude and Lantermann [18] served as measure for perceived social isolation. This instrument has four items (four levels each). An example is: “I am worried to be left behind”. A mean score was computed (ranging from 1 to 4), whereby higher scores reflect higher perceived social isolation. In our current study, Cronbach’s alpha was 0.87 (McDonald’s omega: 0.88) in wave 6. In wave 8, Cronbach’s alpha was also 0.87 (McDonald’s omega: 0.88) in our study.

### Independent variables of interest

First, individuals were asked: “How close is your contact to your neighbours?” [1 = very close; 2 = close; 3 = not really close; 4 = only rare; 5 = no contact]. Moreover, individuals should rate the following statements: (1) “I realise what happens in the neighbourhood”, (2) “I talk with neighbours about what happens in the neighbourhood”, (3) “To a certain extent, I am able to determine what happens in the neighbourhood” [in each case: 1 = strongly agree; 2 = agree; 3 = disagree; 4 = strongly disagree]. These latter three statements may roughly indicate an involvement in neighbourhood activities.

### Covariates

In accordance with former research [6, 7, 19], a wide array of time-varying covariates was selected: Regarding socioeconomic factors, it was adjusted for age (in years), marital status (single; divorced; widowed; married, living separated from spouse; married, living together with spouse), employment situation (employed; retired; other: not employed) and (log) household net equivalence income in Euro. With regard to lifestyle-related factors, it was adjusted for smoking (yes, daily; yes, sometimes; no, not anymore; no, never), alcohol intake (daily; several times a week; once a week; 1–3 times a month; less often; never), and doing sports (daily; several times a week; once a week; 1–3 times a month; less often; never). Regarding health-related factors, it was adjusted for self-rated health (single item ranging from 1 = very good to 5 = very bad), and for a count score for chronic illnesses (ranging from 0 to 11; including: cardiac and circulatory disorders; bad circulation; Joint, bone, spinal and back problems; respiratory problems, asthma, shortness of breath; stomach and intestinal problems; cancer; diabetes; gall bladder, liver or kidney problems; bladder problems; eye problems, vision impairment; ear problems, hearing problems).

Moreover, the time-constant factor sex (men; women) was used for stratifying the FE linear regressions. Furthermore, education (ISCED-97 classification [20], distinguishing between low (0–2), medium (3–4) and high (5–6) education) was used for descriptive purposes.

### Statistical analysis

In a first step, the sample characteristics are shown. Thereafter, multiple linear FE regressions were performed to study the association between changes in our independent variables of interest and changes in our outcomes. In regression analysis, it was adjusted for the time-varying factors listed in the aforementioned section “Covariates”.

A well-known major advantage of FE regressions is that they can deliver consistent estimates under quite weak assumptions. For example, FE regressions produce consistent estimates even when time-constant (observed and unobserved) factors are associated with the predictors. This is in contrast to random-effects regressions which would produce inconsistent estimates in such a case [21].

FE regressions only use changes within individuals over time (e.g., a change in closeness of contact with neighbours within an individual from wave 6 to wave 8). For instance, changes within individuals in loneliness from wave 6 to wave 8 can be examined. For these reasons, only time-varying factors (e.g., self-rated health) can be used as explanatory variables in FE regressions (as main effects). In contrast, time-constant factors (such as sex) cannot be used as main effects. However, it is, for example, possible to stratify the FE regressions by sex. The focus on participants who actually had changes in the independent and the dependent variables over time is not a limitation of the FE strategy, it rather reflects the fact that only a certain proportion of the population actually had such changes over time. In sum, an average treatment effect on the treated is estimated [22].

Our analytical choice was supported by a Sargan–Hansen test (Hausman-test with cluster-robust standard errors): for instance, the Sargan–Hansen statistic was 239.50 (*p* < 0.001) when loneliness served as outcome measure and closeness of contact with neighbors was used as key independent variable. It should be noted that cluster-robust standard errors were calculated [23].

A tool developed by Shaw [24] (“omegacoef”) was used to calculate McDonald’s omega. The statistical significance was determined as *p* value of < 0.05 in this study. Stata 16.1 was used for statistical analyses (Stata Corp., College Station, Texas).

## Results

### Sample characteristics

Sample characteristics for the analytical sample (stratified by sex) are shown in Table 1. In the total analytical sample, average age was 67.4 years (SD: 10.2 years), ranging from 43 to 98 years. Moreover, 50% of the individuals were female and 50% of the individuals had a high education. In sum, about 13.1% of the individuals drank alcohol “daily”, 10.6% of the individuals were daily smokers and 9.9% of the individuals performed sports daily.

**Table 1:** Sample characteristics for the analytical sample stratified by sex (wave 6 and wave 8, pooled)

Variables	Men (*n* = 3344)	Women (*n* = 3344)	Total (*n* = 6688)	*P*-value
	Mean (SD)/N (%)	Mean (SD)/N (%)	Mean (SD)/N (%)	
Age (in years)	68.4 (10.2)	66.4 (10.0)	67.4 (10.2)	< 0.001
Educational level				< 0.001
Low education (ISCED: 0–2)	42 (1.3)	198 (5.9)	240 (3.6)	
Medium education (ISCED: 3–4)	1352 (40.4)	1750 (52.3)	3102 (46.4)	
High education (ISCED: 5–6)	1950 (58.3)	1396 (41.7)	3346 (50.0)	
Marital status				< 0.001
Married, living together with spouse	2625 (78.5)	2115 (63.2)	4740 (70.9)	
Married, living separated from spouse	37 (1.1)	36 (1.1)	73 (1.1)	
Divorced	257 (7.7)	391 (11.7)	648 (9.7)	
Widowed	237 (7.1)	574 (17.2)	811 (12.1)	
Single	188 (5.6)	228 (6.8)	416 (6.2)	
Employment status				< 0.001
Employed	986 (29.5)	1147 (34.3)	2133 (31.9)	
Retired	2248 (67.2)	1937 (57.9)	4185 (62.6)	
Other: Not employed	110 (3.3)	260 (7.8)	370 (5.5)	
Household net equivalent income (in Euro)	2444.5 (5863.9)	2159.4 (1261.3)	2302.0 (4243.3)	< 0.01
Alcohol intake				< 0.001
Daily	638 (19.1)	240 (7.2)	878 (13.1)	
Several times a week	1111 (33.2)	704 (21.1)	1815 (27.1)	
Once a week	526 (15.7)	545 (16.3)	1071 (16.0)	
1–3 × a month	309 (9.2)	496 (14.8)	805 (12.0)	
Less often	506 (15.1)	981 (29.3)	1487 (22.2)	
Never	254 (7.6)	378 (11.3)	632 (9.4)	
Smoking behavior				< 0.001
Yes, daily	350 (10.5)	357 (10.7)	707 (10.6)	
Yes, occassionally	128 (3.8)	88 (2.6)	216 (3.2)	
No, not anymore	1529 (45.7)	1010 (30.2)	2539 (38.0)	
No, never	1337 (40.0)	1889 (56.5)	3226 (48.2)	
Frequency of sports activities				< 0.001
Daily	327 (9.8)	334 (10.0)	661 (9.9)	
Several times a week	964 (28.8)	1044 (31.2)	2008 (30.0)	
Once a week	528 (15.8)	686 (20.5)	1214 (18.2)	
1–3 × a month	213 (6.4)	183 (5.5)	396 (5.9)	
Less often	427 (12.8)	309 (9.2)	736 (11.0)	
Daily	885 (26.5)	788 (23.6)	1673 (25.0)	
Self-rated health (from 1 to 5, with higher values reflecting worse self-rated health)	2.4 (0.8)	2.4 (0.8)	2.4 (0.8)	0.26
Number of chronic conditions (from 0 to 11, with higher values reflecting more chronic conditions)	2.7 (2.0)	2.6 (2.0)	2.6 (2.0)	0.03
Closeness of contact with neighbours				< 0.001
Very close	199 (6.0)	298 (8.9)	497 (7.4)	
Close	1394 (41.7)	1340 (40.1)	2734 (40.9)	
Not really close	1359 (40.6)	1335 (39.9)	2694 (40.3)	
Only rare	358 (10.7)	341 (10.2)	699 (10.5)	
No contact	34 (1.0)	30 (0.9)	64 (1.0)	
Realisation what happens in the neighbourhood				< 0.01
Strongly agree	218 (6.5)	248 (7.4)	466 (7.0)	
Agree	1924 (57.6)	1774 (53.2)	3698 (55.4)	
Disagree	1092 (32.7)	1220 (36.6)	2312 (34.6)	
Strongly disagree	105 (3.1)	92 (2.8)	197 (3.0)	
Talking with neighbours about what happens in the neighbourhood				< 0.001
Strongly agree	300 (9.0)	312 (9.3)	612 (9.2)	
Agree	1715 (51.3)	1547 (46.3)	3262 (48.8)	
Disagree	1113 (33.3)	1250 (37.4)	2363 (35.4)	
Strongly disagree	212 (6.3)	232 (6.9)	444 (6.6)	
Perceived ability to determine what happiness in the neighbourhood				< 0.001
Strongly agree	80 (2.4)	72 (2.2)	152 (2.3)	
Agree	597 (17.9)	446 (13.4)	1043 (15.6)	
Disagree	1708 (51.2)	1631 (48.9)	3339 (50.0)	
Strongly disagree	952 (28.5)	1189 (35.6)	2141 (32.1)	
Loneliness (from 1 to 4, with higher values reflecting higher loneliness)	1.8 (0.5)	1.7 (0.5)	1.8 (0.5)	< 0.001
Perceived social isolation (from 1 to 4, with higher values reflecting higher perceived social isolation)	1.6 (0.6)	1.6 (0.6)	1.6 (0.6)	0.23

Oneway ANOVAs or Chi^2^-tests were conducted, as appropriate (*p*-values)

Additionally, about 41% of the respondents felt a “close” contact to their neighbours, approximately 55% of the respondents agreed that they realized what happens in their neighbourhood, nearly 49% of the respondents agreed that they talked with neighbours about what happens in the neighbourhood, and 50% of the respondents disagreed that they have an ability to determine what happens in the neighbourhood. Furthermore, the average loneliness score was 1.8 (SD: 0.5) and the average perceived social isolation score was 1.6 (SD: 0.6). More details are given in Table 1. It should be noted that the pairwise correlation between loneliness and perceived social isolation was *r* = 0.50 in wave 6 (wave 8: *r* = 0.51), *p* < 0.001 (both waves).

### Regression analysis

The results of linear FE regressions stratified by sex are shown in Table 2 (among men) and in Table 3 (among women), respectively. Prior to FE regression analysis, we used the Stata commands “xttrans” (for continuous variables) and “xttab” (for categorical variables) to check whether there is enough within-variation in the data to obtain precise estimates (results not shown, but available upon request). Our respective results showed that there is enough within-variation. Thus, in all FE regressions, it was adjusted for these sociodemographic, lifestyle-related and health-related time-varying factors: age, marital status, employment situation, income, smoking behavior, alcohol intake, sports activities, self-rated health and chronic illnesses.

**Table 2:** Neighbourhood cohesion and loneliness as well as perceived social isolation among men

	Loneliness	Perceived social isolation	Loneliness	Perceived social isolation	Loneliness	Perceived social isolation	Loneliness	Perceived social isolation
Closeness of contact with neighbours:—Close (Ref.:—Very close)	0.11**	0.04						
	(0.04)	(0.04)						
-Not really close	0.15**	0.11*						
	(0.05)	(0.05)						
-Only rare	0.19***	0.20**						
	(0.06)	(0.07)						
-No contact	0.23*	0.10						
	(0.10)	(0.16)						
Realisation what happens in the neighborhood:—Agree (Ref.: Strongly agree)			0.05	0.03				
			(0.04)	(0.04)				
-Disagree			0.07	0.03				
			(0.04)	(0.05)				
-Strongly disagree			− 0.03	− 0.07				
			(0.08)	(0.09)				
Talking with neighbours about what happens in the neighborhood:—Agree (Ref.: Strongly agree)					0.03	0.00		
					(0.03)	(0.04)		
-Disagree					0.01	0.01		
					(0.04)	(0.04)		
-Strongly disagree					0.03	0.01		
					(0.06)	(0.07)		
Perceived ability to determine what happiness in the neighbourhood:—gree (Ref.: Strongly agree)							0.07	0.06
							(0.07)	(0.06)
-Disagree							0.07	0.06
							(0.07)	(0.07)
-Strongly disagree							0.01	0.03
							(0.07)	(0.07)
Potential confounders^†^	✓	✓	✓	✓	✓	✓	✓	✓
Observations	3358	3344	3360	3346	3358	3354	3345	3342
Individuals	1679	1672	1680	1673	1679	1677	1677	1671
R^2^	0.04	0.04	0.03	0.03	0.03	0.03	0.04	0.03

Results of linear FE regressions

Unstandardized beta coefficients are shown. Cluster-robust standard errors are shown in parentheses

***p < 0.001

**p < 0.01

*p < 0.05

^+^*p* < 0.10

^†^Potential time-varying covariates include age, marital status, employment status, (log) household equivalence net income, smoking status, alcohol intake, frequency of sports activities, self-rated health and the number of chronic conditions

**Table 3:** Neighbourhood cohesion and loneliness as well as perceived social isolation among women

	Loneliness	Perceived social isolation	Loneliness	Perceived social isolation	Loneliness	Perceived social isolation	Loneliness	Perceived social isolation
Closeness of contact with neighbours:—Close (Ref.:—Very close)	− 0.05	− 0.03						
	(0.03)	(0.04)						
-Not really close	− 0.03	0.02						
	(0.04)	(0.05)						
-Only rare	− 0.00	0.09						
	(0.05)	(0.06)						
-No contact	0.10	0.01						
	(0.10)	(0.17)						
Realisation what happens in the neighbourhood:—Agree (Ref.: Strongly agree)			0.07	− 0.01				
			(0.04)	(0.05)				
-Disagree			0.08^+^	0.07				
			(0.04)	(0.06)				
-Strongly disagree			0.18**	0.20*				
			(0.07)	(0.10)				
Talking with neighbours about what happens in the neighbourhood:—Agree (Ref.: Strongly agree)					0.09**	0.07^+^		
					(0.03)	(0.04)		
-Disagree					0.09*	0.09*		
					(0.04)	(0.04)		
-Strongly disagree					0.10*	0.14*		
					(0.05)	(0.06)		
Perceived ability to determine what happiness in the neighbourhood:—Agree (Ref.: Strongly agree)							0.00	0.08
							(0.07)	(0.07)
-Disagree							0.02	0.16*
							(0.07)	(0.08)
-Strongly disagree							− 0.01	0.17*
							(0.08)	(0.08)
Potential confounders^†^	✓	✓	✓	✓	✓	✓	✓	✓
Observations	3358	3344	3348	3334	3362	3348	3358	3342
Individuals	1679	1672	1674	1667	1681	1674	1679	1671
*R*^2^	0.04	0.03	0.04	0.02	0.04	0.02	0.03	0.03

Results of linear FE regressions

Unstandardized beta coefficients are shown. Cluster-robust standard errors are shown in parentheses

***p < 0.001

**p < 0.01

*p < 0.05

^+^*p* < 0.10

^†^Potential time-varying covariates include age, marital status, employment status, (log) household equivalence net income, smoking status, alcohol intake, frequency of sports activities, self-rated health and the number of chronic conditions

In men (Table 2), decreases in closeness of contact with neighbours were associated with increases in loneliness (e.g., from “very close” contact to “not really close”: *β* = 0.19, *p* < 0.001). Moreover, decreases in closeness of contact with neighbours were associated with increases in perceived social isolation (e.g., from “very close” contact to “only rare”: *β* = 0.20, *p* < 0.01). In contrast, the other neighbourhood-related factors were neither associated with loneliness nor with perceived social isolation.

In women (Table 3), changes in closeness of contact with neighbours were not significantly associated with both loneliness and perceived social isolation. In contrast, a decrease in agreement with the statement “I realise what happens in the neighbourhood” was associated with an increase in both loneliness (from “strongly agree” to “strongly disagree”: *β* = 0.18, *p* < 0.01) and perceived social isolation (from “strongly agree” to “strongly disagree”: *β* = 0.20, *p* < 0.05). Similarly, a decrease in agreement with the statement “I talk with neighbours about what happens in the neighbourhood” was associated with an increase in both loneliness (e.g., from “strongly agree” to “disagree”: *β* = 0.09, *p* < 0.05) and perceived social isolation (e.g., from “strongly agree” to “disagree”: *β* = 0.09, *p* < 0.05). Additionally, a decrease in agreement with the statement “To a certain extent, I am able to determine what happens in the neighbourhood” was associated with an increase in perceived social isolation (e.g., from “strongly agree” to “disagree”: *β* = 0.16, *p* < 0.05).

## Discussion

Based on data, from a large, longitudinal nationally representative sample, our aim was to examine the association between neighbourhood cohesion and loneliness as well as perceived social isolation using a longitudinal approach (stratified by sex). Our key findings were as follows: FE regressions showed that decreases in closeness of contact with neighbours were associated with increases in loneliness and perceived social isolation levels among men, but not women. In contrast, decreases in different indicators of involvement in neighbourhood activities were associated with increases in loneliness and perceived social isolation levels among women, but not men. This current longitudinal study using data prior and during the COVID-19 pandemic extends our knowledge mainly based on cross-sectional studies prior to the pandemic.

The fact that changes in the pure closeness of contact with neighbours were significantly associated with the outcomes only among men, but not women may be explained as follows: the closeness of contact with neighbours may reflect factors such as actual friendships among men (e.g., to barbecue together or watch sports together). This closeness can potentially contribute to feelings of belonging in men. Additionally, men may more heavily rely on friendships from the neighbourhood. Women, in contrast, could have social networks from very different life areas and may not rely on closeness of contact with neighbours for their social activities.

Another possible explanation is that the closeness of contact may mainly refer to the quantity, whereas the things that happen in the neighborhood may mainly refer to the quality of the relationship. Older men probably have smaller social networks compared to women [25]. If the contact with neighbors decreases, this may have a noticeable effect among older men. Women, as outlined above, may more easily compensate for this with other friends [26].

In contrast to the closeness of contact with neighbours, things that happen in the neighborhood (in terms of realization, communicating and the ability to determine it) are important for loneliness and perceived social isolation among women, but not men. For men, for example, these happenings in the neighbourhood might be of less interest, whereas, for example, communicating about the factors happening in the neighbourhood may contribute to feelings of inclusion, embeddedness and belongingness in women. This could also be due to the fact that—at least in these birth cohorts—middle-aged and older women may spend a greater proportion of their time in their neighbourhood (e.g., due to differences in working hours between women and men). It would be interesting to explore in future studies whether these findings can be replicated in younger birth cohorts. For example, due to the increasing labor force participation of women in many countries, somewhat different results appear to be possible.

Some strengths and limitations are worth keeping in mind when interpreting our current findings. First, a large, nationally representative sample was used. Moreover, the outcomes were assessed using established and valid tools. Additionally, different items were used to assess neighbourhood cohesion. Furthermore, longitudinal data were used. Because FE regressions were used, the problem of unobserved heterogeneity—a key challenge when using observational data—was markedly reduced. It should be acknowledged that a small sample selection bias has been identified in the DEAS study [16]. For example, participation rates are, for example, lower among individuals aged 70–85 years or among individuals living in large cities. However, such effects are small and the distribution of the sociodemographic characteristics is very close to the distribution in Germany [16].

## Conclusion

Changes in neighbourhood factors are differently associated with loneliness and perceived social isolation among middle-aged and older women and men. Gender-specific efforts to avoid loneliness and social isolation are, therefore, needed. With regard to future research, studies from other countries are required to confirm our findings. Moreover, upcoming research should elucidate the underlying mechanisms.

## Data Availability

The data used in this study are third-party data. The anonymized data sets of the DEAS (1996, 2002, 2008, 2011, 2014, 2017, 2020, 2020/2021) are available for secondary analysis. The data have been made available to scientists at universities and research institutes exclusively for scientific purposes. The use of data is subject to written data protection agreements. Microdata of the German Ageing Survey (DEAS) are available free of charge to scientific researchers for non-profitable purposes. The FDZ-DZA provides access and support to scholars interested in using DEAS for their research. However, for reasons of data protection, signing a data distribution contract is required before data can be obtained. For further information on the data distribution contract, please see https://www.dza.de/en/research/fdz/access-to-data/formular-deas-en-english (accessed on 10 November 2022).
